# Spatial variation of sediment mineralization supports differential CO_2_ emissions from a tropical hydroelectric reservoir

**DOI:** 10.3389/fmicb.2013.00101

**Published:** 2013-04-30

**Authors:** Simone J. Cardoso, Luciana O. Vidal, Raquel F. Mendonça, Lars J. Tranvik, Sebastian Sobek, Roland Fábio

**Affiliations:** ^1^Laboratory of Aquatic Ecology, Federal University of Juiz de ForaJuiz de Fora, Brazil; ^2^Department of Ecology, Program of Post Graduation in Ecology, Biology Institute, Federal University of Rio de JaneiroRio de Janeiro, Brazil; ^3^Department of Ecology and Evolution, Uppsala UniversityUppsala, Sweden

**Keywords:** freshwater sediment, carbon mineralization, stable isotopes, *p*CO_2_, heterotrophic bacteria, hydroelectric reservoir, Cerrado, Brazil

## Abstract

Substantial amounts of organic matter (OM) from terrestrial ecosystems are buried as sediments in inland waters. It is still unclear to what extent this OM constitutes a sink of carbon, and how much of it is returned to the atmosphere upon mineralization to carbon dioxide (CO_2_). The construction of reservoirs affects the carbon cycle by increasing OM sedimentation at the regional scale. In this study we determine the OM mineralization in the sediment of three zones (river, transition, and dam) of a tropical hydroelectric reservoir in Brazil as well as identify the composition of the carbon pool available for mineralization. We measured sediment organic carbon mineralization rates and related them to the composition of the OM, bacterial abundance and *p*CO_2_ of the surface water of the reservoir. Terrestrial OM was an important substrate for the mineralization. In the river and transition zones most of the OM was allochthonous (56 and 48%, respectively) while the dam zone had the lowest allochthonous contribution (7%). The highest mineralization rates were found in the transition zone (154.80 ± 33.50 mg C m^-^^2^ d^-^^1^) and the lowest in the dam (51.60 ± 26.80 mg C m^-^^2^ d^-^^1^). Moreover, mineralization rates were significantly related to bacterial abundance (*r*^2^ = 0.50, *p* < 0.001) and *p*CO_2_ in the surface water of the reservoir (*r*^2^ = 0.73, *p* < 0.001). The results indicate that allochthonous OM has different contributions to sediment mineralization in the three zones of the reservoir. Further, the sediment mineralization, mediated by heterotrophic bacteria metabolism, significantly contributes to CO_2_ supersaturation in the water column, resulting in higher *p*CO_2_ in the river and transition zones in comparison with the dam zone, affecting greenhouse gas emission estimations from hydroelectric reservoirs.

## INTRODUCTION

Significant efforts have been made to understand the carbon fluxes into and out of inland waters. However, discerning the pathways of carbon flow, especially related to aquatic sediments, remains uncertain (Cole et al., 2007). For instance, the relationship between the allochthonous carbon loaded to aquatic ecosystems and the portion either mineralized or buried in sediments has yet to be better quantified (Battin et al., 2009; Sobek et al., 2011). Inland waters (e.g., lakes and reservoirs) receive large amounts of allochthonous organic matter (OM) transported from the surrounding watershed, part of which is sedimented. Once in the sediment, OM can have three major fates: (i) mineralization by aerobic or anaerobic bacteria and release back to water column as CO_2_ and CH_4_; (ii) re-suspension and mineralization in the water column; or (iii) burial in the sediment. The relative importance of each of these three processes is system-specific and influences the overall role of a system as net sink or source of carbon as greenhouse gases (GHG) to the atmosphere (Mendonça et al., 2012a). The limited knowledge of the lateral transport of carbon from soils, and its fates in lakes and reservoirs results in incomplete understanding of the terrestrial carbon balance (Tranvik et al., 2009).

Hydroelectric reservoirs are man-made freshwater ecosystems that can substantially alter regional and global carbon balance. During the first years after impounding, the mineralization of flooded vegetation and soils cause significant emissions of GHG to the atmosphere (Mendonça et al., 2012b). Growing demand for energy has motivated the construction of hydroelectric reservoirs worldwide. There are approximately 45,000 large hydroelectric reservoirs in operation in the world (World Commission on Dams [WCD], 2000), with a total surface area of about 350,000 km^2^ (Barros et al., 2011). In Brazil, the increasing number of hydroelectric reservoirs in pristine areas (e.g., Amazon and Pantanal) has generated controversy among local people and among the scientific community worldwide (Fearnside, 2006; Sousa Júnior and Reid, 2010). One source of dispute is the effect of river damming on aquatic GHG emissions. In this context, estimating inputs and outputs of OM in hydroelectric reservoirs is important in order to better understand the impacts of these systems on the environment and to support regional and global evaluations of the environmental costs and benefits of hydroelectric energy generation.

In tropical freshwater ecosystems, sediment OM is mineralized by aerobic and anaerobic respiration and fermentation resulting in the production of CO_2_ or CH_4_. The occurrence of aerobic or anaerobic processes in the sediment is regulated by the availability of electron acceptors (e.g., oxygen, nitrate, manganese, iron, and sulfate; Fenchel et al., 1998). Especially in the case of reservoirs, those processes are also regulated by mixing regimes, thermal structure of the water column and productivity (Kalff, 2002) that vary along a longitudinal gradient, resulting in the establishment of different zones. Those zones are mainly the area close to the river inflow, the transitional area between the river and the dam of the reservoir, and a lacustrine zone directly influenced by the dam of the reservoir (Thornton et al., 1990; De Junet et al., 2009). Additionally, the magnitude of the carbon degradation across reservoir zonation can be related to the amount and quality of organic carbon (OC) available (Conrad et al., 2010, 2011), plankton metabolism (Forbes et al., 2012), oxygen concentrations (Sobek et al., 2009) and the temperature of the water overlaying the sediment (Gudasz et al., 2010). Despite the mineralization processes in the sediment are gaining more attention in aquatic ecosystems (Algesten et al., 2005; Battin et al., 2009), studies considering the mineralization in the sediment of hydroelectric reservoirs are still rare, particularly in tropical areas.

In this study, our goal was to determine the OM mineralization in the sediment of three zones (river, transition, and dam) of a tropical hydroelectric reservoir in Brazil as well as characterize the source of the carbon pool available for mineralization. The results indicate that OM mineralization in the sediment vary along the different zones of the reservoir. It is mostly influenced by the allochthonous carbon pool and significantly contributes to CO_2_ supersaturation in the water column affecting GHG emissions from the system.

## MATERIALS AND METHODS

### STUDY AREA DESCRIPTION

This study was conducted in the Manso Reservoir (14°52^′^ S, 55°46^′^ W) in January of 2009. The reservoir is located in southeastern Brazil, in the Cerrado (savannah-type) biome. It is a large (360 km^2^) and deep (maximum depth 40 m) hydroelectric reservoir that was formed by damming the Manso River in 1999. The Manso River is a tributary of the Cuiabá River, one of the main drainages of the Pantanal, an important wetland in Brazil. Water residence time in the Manso Reservoir is approximately 2.5 years, and the turbine intake is located in the epilimnion (Roland et al., 2010). The reservoir is mesotrophic with total phosphorus (TP) concentrations ranges of 25–37 μgL^-^^1^, total nitrogen (TN) concentrations ranges of 530–800 μgL^-^^1^, and chlorophyll-a concentrations ranges of 2–6 μgL^-^^1^ (Rangel et al., 2012). During the samplings the bottom water of the reservoir was oxygenated and neutral with dissolved oxygen (DO) concentrations varying from 4 (dam) to 7 (river and transition) mg L^-^^1^ and pH of 7 ± 0.3 (mean and standard deviation).

### SAMPLING

**FIGURE 1:**
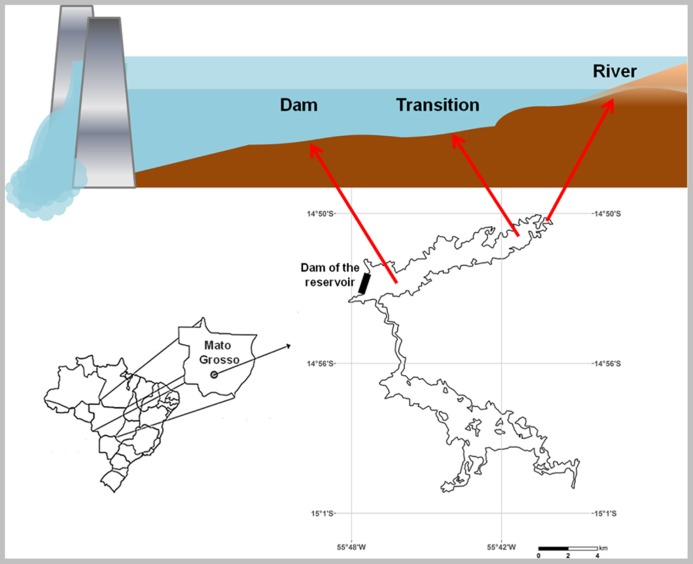
**Map of the study area showing the different sampling zones of the Manso reservoir: river (14 m depth) – an area close to the inflow of the Manso River; transition (11 m depth) – an intermediate area between the river inflow and the dam, and dam (27 m depth) – a lacustrine area directly influenced by the dam of the reservoir**.

Water samples for carbon stable isotope signature (δ^13^C) of the particulate organic matter (POM) were taken from the surface, middle and bottom of the water column in each of the different zones. Sediment cores were collected using a gravitational sediment sampler (Kajak, modified by Ambühl and Bührer, 1975) in the same three zones of one arm of the Manso Reservoir. These zones were river (14 m depth) – an area close to the inflow of the Manso River; transition (11 m depth) – an intermediate area between the river inflow and the dam, and dam (27 m depth) – a lacustrine area directly influenced by the dam of the reservoir (**Figure 1**). From each zone five sediment cores were taken. The upper 10 cm was transferred to incubation cores (54 mm inner diameter) without visible disturbance of the sediment structure. The incubation cores were then totally filled with water from the bottom of the sampling sites collected with a Van Dorn device. The incubation cores were equipped with internal magnetic stirring devices to allow mixing of the overlying water without disturbing the sediment surface. Aliquots of the overlying water were taken from each core to determine initial concentrations of dissolved inorganic carbon (DIC), *p*CO_2_, pH, temperature and bacterial abundances. The cores were then sealed without headspace with an expandable polyvinyl chloride (PVC) stopper fitted with double o-rings and outlet tubing. The cores were maintained under *in situ* temperature and in the dark, to avoid primary production. Samples of water were taken after 12, 24, 72, and 96 h of incubation by pushing the PVC stopper downwards in the core and simultaneously filling plastic syringes. All cores were incubated in oxic conditions with initial DO concentration of 6.0 ± 0.3 mg L^-^^1^ and pH 7 ± 0.3. As the mineralization of OM in the sediment comprises different metabolic processes, including aerobic oxidation and different anaerobic oxidation that lead to the release of CO_2_ in the water, we measured the net effect of all these processes, represented by the DIC production in the cores. Sediment samples from the top 2 cm, representing the layer immediately in contact with water, and 10 cm, the limit layer of significant bacterial activity in the sediment, where taken at the beginning (from extra cores) and at the end of the incubation (from cores used for the incubation) to analyze the concentrations of OM, total carbon (TC), TN, TP, and δ^13^C isotope composition.

### ANALYTICAL METHODS

Water samples from the cores were analyzed for pH using a pH meter (Micronal B474), DO concentrations with a Clark-type oxygen sensor coupled to a picoamperimeter (Unisense^©^, model PA 2000) and DIC following sodium persulfate digestion on a Tekmar-Dohrmann TC analyzer (model Phoenix 8000). The *p*CO_2_ was measured both in the water overlying the cores and in the water surface of the reservoir using an infrared gas analyzer (IRGA- environmental gas monitor EGM-4; PP Systems). Measurements of *p*CO_2_ were made directly using the headspace equilibrium method (Hesslein et al., 1991; Cole et al., 1994). Fifteen mL of atmospheric air was equilibrated with 20 mL of water by vigorous shaking for 1 min (Cole and Caraco, 1998). The headspace gas was transferred to a plastic syringe, and the concentration of CO_2_ was immediately measured on the IRGA. Bacterial abundance from the overlying water of the sediment was estimated by direct counting using the acridine orange method (Hobbie et al., 1977) under an Olympus BX60 fluorescence microscope.

Sediment samples of OM were analyzed by loss on ignition and TP by the colorimetric method according to Carmouze (1994). Concentrations of TC and TN in the sediment were analyzed according to Mackereth et al. (1978) using a PerkinElmer analyzer. Sediment particle sizes were quantified using a Malvern laser diffraction particle size analyzer. For the determination of water content and porosity, subsamples of fresh sediment were weighed in ceramic vessels and their weight loss recorded after heating for 4 days at 60°C (Dalsgaard et al., 2000).

The particulate organic carbon (POC) concentration and the stable isotope ratios of POC and sediment samples were analyzed using a gas isotope ratio mass spectrometer (Delta Plus, Finnigan Mat). One replicate sample and standards were analyzed after every set of 10 samples. Carbon isotopic ratios were expressed using delta notation as the ratio of the heavy to the light isotope (δ^13^C) over the ratio of the heavy to the light isotope of the international standards δ^13^C Pee Dee Belemnite, respectively. Isotope ratios of the OM samples were used to determine the allochthonous contribution to the OM pool (Gu et al., 2011). We estimated the upper and lower limits for the contribution of each carbon source (autochthonous and allochthonous) to mineralization in the different zones of the reservoir by using a two-source isotope mixing model (Fry, 2006): terrestrial material and algae. Macrophytes, were excluded from the sources due to the low abundance in the sampled sites.

The end members (OM sources) for the two-source mixing model were determined as follows: during the sampling period there was a bloom of algae in the reservoir. POM samples were acquired by passing subsurface water samples through a glass fiber filter (0.7 μm porosity). Subsurface water samples were also used for phytoplankton analysis. The phytoplankton analysis showed dominance in abundance and composition (90%) of the cyanobacteria *Microcystis aeruginosa *in the POM samples. The δ^13^C of these samples, -30.10‰, was used as the pelagic end member in the mixing model. During the samplings there was no visual confirmation of macrophytes along the reservoir. As the rivers are known to have a predominant contribution of allochthonous material in the surface waters (Kalff, 2002) we used POM samples from the surface water of this zone for isotope analysis. The δ^13^C value of these samples (-19.26‰) was used as the allochthonous end member in the mixing model.

Differences in OC mineralization, *p*CO_2_ in the incubation chambers and the surface waters, and bacterial abundance in the sediment among zones was tested using ANOVA, followed by Tukey’s *post hoc* test performed in SigmaPlot 11.0.

## RESULTS

**FIGURE 2:**
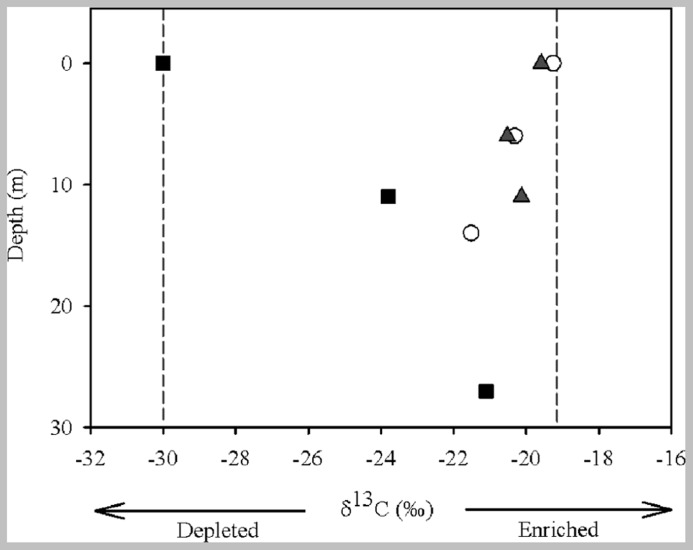
**Carbon isotope composition of the POM from the water sampled at different depths (surface, middle, and bottom) and in different zones (river = open circle, transition = gray triangle, and dam = black square) of the Manso Reservoir**. Light gray lines represent the end members (allochthonous: δ^13^C -19.26‰ and autochthonous: δ^13^C -30.10‰).

**FIGURE 3:**
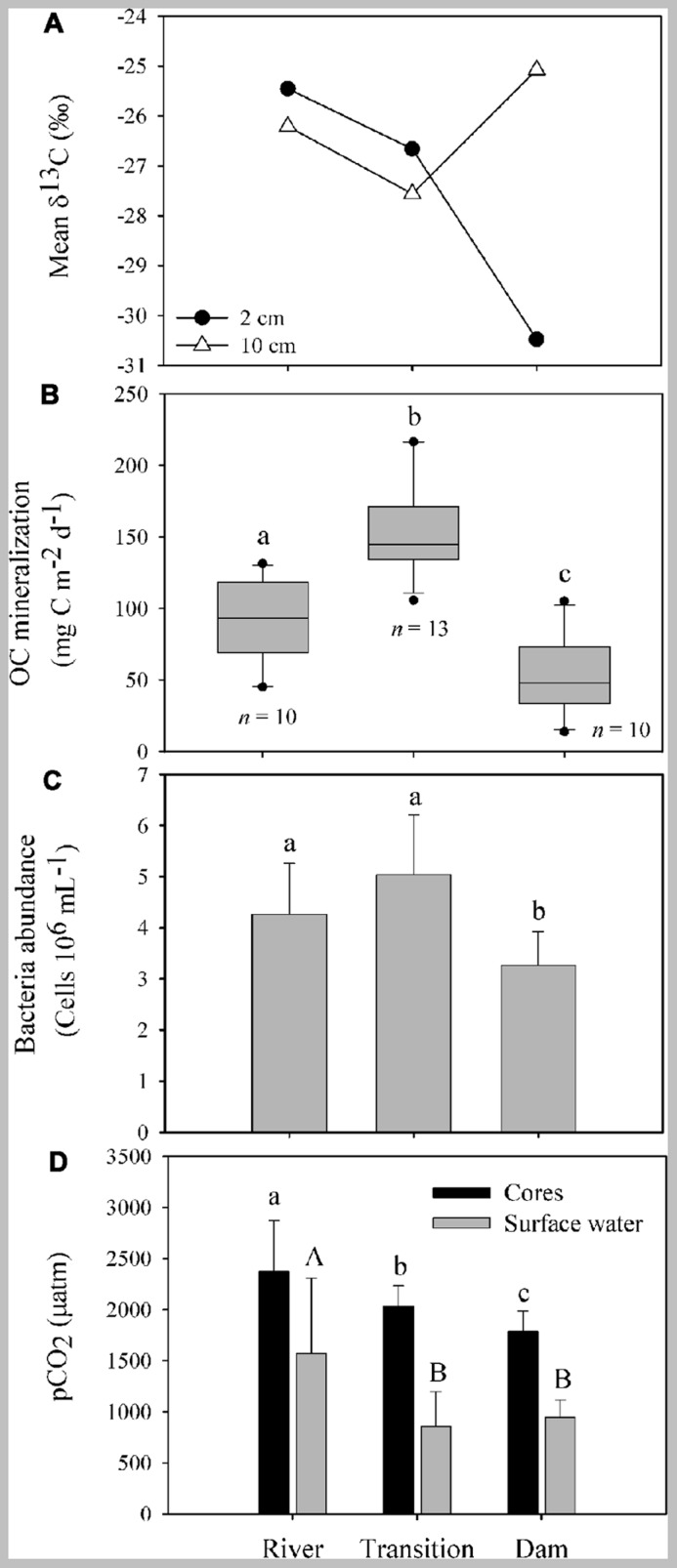
**Characteristics of OM composition, OC mineralization, bacteria abundance and partial pressure of CO_2_ in the sediment of different zones of the Manso Reservoir**. **(A)** Carbon isotope composition of the 2 and 10 cm of sediments sampled in different zones of the Manso Reservoir. **(B)** OC mineralization rates measured in the overlying water of sediment core incubations. The boundary of the box closest to zero indicates the 25th percentile, a line with the box marks the median, and the boundary of the box farthest from zero indicates the 75th percentile. Error bars above and below the box indicate the 90th and 10th percentiles and dark circles represent the outliers. **(C)** Mean and standard deviation of bacterioplankton abundance measured in the overlaying water of the sediment cores incubations. Small letters represent the differences between the *p*CO_2_ from overlaying water of the sediment cores incubations (black bars) and capital letters represent the differences between the *p*CO_2_ measure on the surface water of the reservoir (gray bars).** (D)** Means and standard deviations of partial pressure of CO_2_ (*p*CO_2_) from the overlaying water of the incubation cores (black bars) and in the surface water of the reservoir (gray bars; data from Roland et al., 2010). In all panels different letters represent significant differences (one-way ANOVA, Tukey’s *post hoc*, *p* < 0.001).

The suspended POC stable isotope composition varied over depth and between the different zones (river, transition, and dam; **Figure 2**). In the river, δ^13^C values decreased from -19.26 (surface) to -20.32 (middle), and to -21.51‰ (bottom). An opposite pattern was found for δ^13^C values in the dam -30.10 (surface), -23.70 (middle), and -21.10‰ (bottom). The different zones of the reservoir had similar concentrations of OM and nutrients (TC, TN, and TP) in the first two centimeters of sediment (**Table 1**). Although the concentration of OM was similar, the composition of the pools differed (**Figure 3A**). The river zone sediment was mostly allochthonous, with allochthonous contributions of 56 (2 cm) and 48% (10 cm) and δ^13^C of -25.50 and -26.20‰, respectively. In the transition zone, allochthonous contributions to the OM pool were 41% (2 cm) with δ^13^C of -26.70‰ and 35% (10 cm) with δ^13^C of -27.60‰. The dam zone was the only zone with distinct autochthonous composition. The 2 cm depth had a prominent phytoplankton component (-30.48‰) and the estimated allochthonous contribution was 7% to the OM pool. The 10 cm depth sediment was however, mostly allochthonous (60% of the OM pool, δ^13^C was – 25.10‰). Field observations also revealed the presence of blooms of the cyanobacteria *M. aeruginosa* near the dam, which may have contributed to the aquatic signature of the OM pool in the first 2 cm of the sediment. In all zones the 2 cm sediment depth grain sizes were classified largely as fine (8–16 μm) and very fine silt (4–8 μm) classes. Porosity ranged from 0.70 ± 0.10 at the 2 cm depth to 0.52 ± 0.10 at the 10 cm for all zones.

**Table 1:** Organic matter (OM), total carbon (TC), total nitrogen (TN), ratio between TC and TN (C/N), total phosphorus (TP) concentrations at 2 and 10 cm of sediments sampled in the different zones of the Manso reservoir.

System	Depth (cm)	OM (% DW)	TC (% DW)	TN (% DW)	C/N (by atom)	TP (mg g DW^-1^)
River	2	9.97	5.03	0.39	12.90	0.07
	10	14.64	2.83	0.17	16.65	0.08
Transition	2	10.73	2.25	0.18	12.50	0.07
	10	15.82	5.56	0.32	17.38	0.07
Dam	2	11.31	2.90	0.22	13.18	0.08
	10	2.35	0.59	0.04	14.75	0.04

**FIGURE 4:**
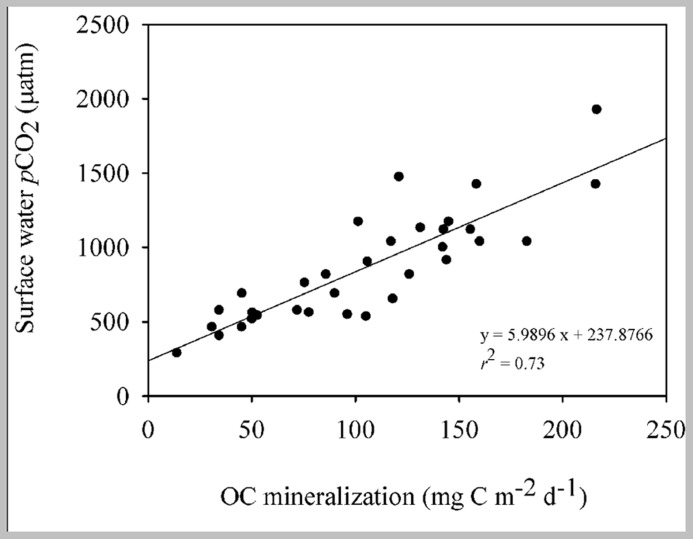
**Linear regression describing the relationship between OC mineralization rates in the sediment and the partial pressure of CO_2_ (*p*CO_**2**_) in the surface water of the reservoir (*n* = 33; *p* < 0.001)**.

The organic carbon mineralization was different among the three zones with the highest average values in the transition (154.80 ± 33.50 mg C m^-^^2^ d^-^^1^), followed by the river (91.30 ± 28.70 mg C m^-^^2^ d^-^^1^) and the dam (51.60 ± 26.80 mg C m^-^^2^ d^-^^1^; **Figure 3B**). The bacterial abundance followed the same pattern as the mineralization rates as it was higher in the transition (5.10 ± 1.20 cells 10^6^ mL^-^^1^; **Figure 3C**) and the river (4.30 ± 1 cells 10^6^ mL^-^^1^). While no significant differences between bacterial abundance in the transition and river were found (*p* = 0.44), both zones were statistically different from the dam (3.30 ± 0.70; *p* < 0.001). The *p*CO_2_ in the overlying water of the sediment was significantly different among the three zones (*p* < 0.001), gradually decreasing from the river to the dam, with highest values at the river (2370.60 ± 500 μatm) relative to the transition (2035.20 ± 10 μatm) and dam (1785.20 ± 100 μatm; **Figure 3D**). The *p*CO_2_ in the surface water along the reservoir (data from Roland et al., 2010) also showed high values of *p*CO_2_ on the river, but, with no statistical differences in the other zones (*p* = 0.82; **Figure 3D**). The organic carbon mineralization in the sediment was also positively related to the *p*CO_2_ in the surface water of the reservoir (*p* < 0.001, *r*^2^ = 0.73; **Figure 4**).

**FIGURE 5:**
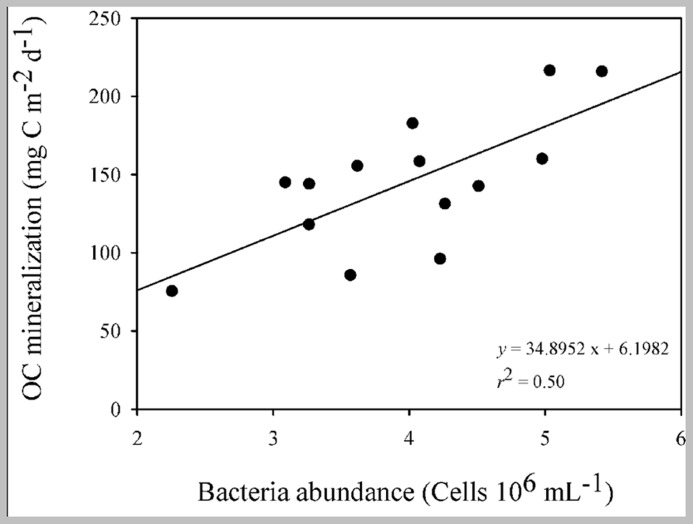
**Linear regression describing the relationship between bacteria abundance and OC mineralization rates from the overlying water of the incubation cores over time (*n* = 18; *p* < 0.001)**.

The bacterial abundance was positively related to OC mineralization (*p* < 0.001, *r*^2^ = 0.50; **Figure 5**) in the overlaying water of the sediment cores.

## DISCUSSION

The OC mineralization rates in the sediment indicated that the allochthonous OM supply was an important fuel to the metabolism in the sediment of different zones of the Manso Reservoir. The stable isotope analysis of POM from different water column depths suggested different pools of OC in the zones, with isotopic signatures more enriched (allochthonous) in the river and transition and more depleted (autochthonous) in the dam. The δ^13^C of POM of the first 2 cm of sediment followed the same pattern of the POM sources in the water, and reflected recent processes showing more ^13^C-enriched in the river and transition zones, probably a result of the terrestrial material coming from the littoral areas, and more depleted in the dam, where the effect of littoral areas is lower and there is higher contribution of primary producers (e.g., phytoplankton) in relation to the other zones (Kalff, 2002; Tremblay et al., 2004). Degradation of POM during sinking along the water column and in surficial sediment layers may cause changes in the isotopic signature (Meyers and Eadie, 1993). Although, for a lake sediment of both autochthonous and allochthonous origin, the δ ^13^C has been reported to increase by 0.4–1.5‰ during the initial degradation, with only minor changes after that period (Gälman et al., 2009). This fractionation during initial degradation is comparatively small and will therefore only introduce a minor error in the calculated proportions of allochthonous POM. We observed a change in the POM signatures in the samples from the different water column depths of the dam zone, with more enriched signature in the bottom (27 m). However, the top 2 cm of sediment of the dam zone had a more depleted signature. In this case, the depleted isotopic signature of the sediment may be related to past plankton blooms and previous mixing events that could have caused higher deposition of the phytoplankton from the water column. The 10 cm sediment layer was mostly allochthonous in the different zones, once the isotopically light phytoplankton debris may be preferentially degraded along the time. This layer can be seen as a reflection of previous conditions in the reservoir and is composed by older OM than the 2 cm.

The sediment sampled in 2 and 10 cm layers was mainly composed of fine and very fine silt, rich in carbon and nitrogen and poor in phosphorus compared to other water bodies (Lennon and Pfaff, 2005), what meets the mesotrophic condition of the system (Rangel et al., 2012). By the time of the survey, the Manso Reservoir had been in operation for 10 years and along its whole area, mainly in the dam, it was still possible to find a large number of dead trees remaining from the beginning of the impoundment. We believe the dead trees probably no longer contribute to mineralization in the sediment anymore, since they died more than 10 years ago and the remaining biomass was still there because of their very slow decay (Tremblay et al., 2004). Also, there was no visual contribution of aquatic macrophytes during the sampling, what indicates that phytoplankton and terrestrial material were, respectively, the most important autochthonous and allochthonous sources of carbon for the sediment mineralization during the sampling period.

A gradient of allochthonous OM contribution was found in the top 2 cm of sediment along the zones, with the greatest contribution in the river zone (56%), less in the transition zone (48%), and even less in the dam zone (7%). The OC mineralization followed the same pattern and increased with increasing inputs of allochthonous OM in the sediment. However, it is important to highlight that the origin and quality of the OM in the different zones may not be the same. For instance, the literature points out that allochthonous carbon reaching the rivers is mostly composed of terrestrial OM coming from the watershed, and this OM is more difficult to degrade and assimilate by aquatic bacteria (Kritzberg et al., 2005) and fungi (Gessner et al., 2007) communities, while the OM in the lacustrine area of the reservoir is more labile and is mostly released by primary producers in the water column (Tremblay et al., 2004; Forbes et al., 2012). However, in the river and in the transition zones, in spite of the OM be less degradable, there is higher OM deposition in comparison with the dam zone: as water flows become less turbulent upon entry of the river water into the reservoir, suspended particles will be rapidly deposited in the river zone and transition zone. We hypothesize that the higher amount of allochthonous OM deposition in the river and transition zones compensates for the low degradability of the allochthonous OM, because, with more OM available in the sediment, more OC is delivered to sediment bacteria and fungi, promoting higher OC mineralization rates in those zones. In addition, it is important to consider that OM sedimentation can also be influenced by the reservoir hydrodynamics, making it more difficult to trace OM source with a two source mixing model.

The Manso Reservoir is a typical tropical hydroelectric reservoir with a marked longitudinal gradient forming the river, the transition and the dam zones addressed in this study and first proposed by the reservoir zonation theory (see Thornton et al., 1990). The Manso Reservoir has complex hydrology, influenced by seasonal climatic conditions and by the river Manso dynamics. During the summer season (from late December to early March), when our sampling was performed, the Manso Reservoir is mostly stratified (Pacheco et al., 2011). However, sporadic heavy rain may occur in the region, mixing the entire water column of the reservoir. Studies made in the Manso reservoir during the same period of our sampling (Assireu et al., 2011; Pacheco et al., 2011) reported a plunge inflow of the river Manso in the reservoir, allocating the river at the hypolimnion level. This inflow of the Manso River in the reservoir highlights possible interactions between the river water and the POM pools in hypolimnion of the dam zone. In fact, it supports the similar δ^13^C signatures of the POM found in the hypolimnion of dam (27 m) and the water samples of the river and the transition zones.

Heterotrophic bacteria were an important link between the allochthonous OM, the OC metabolism in the sediment and the release of carbon from sediment to water in the reservoir. In our survey we found a positive relationship between bacteria abundances and the OC mineralization rates (**Figure 5**). Similar relationships between bacterial abundance and OC mineralization in the sediment were found in a Chinese lake with different trophic conditions, where increasing contributions of allochthonous OC enhanced bacteria density, biomass, and diversity (Bai et al., 2012). Also, a positive relationship between OC mineralization in the sediments and bacteria biomass was found in a survey of eight boreal lakes (Gudasz et al., 2012). Those findings reinforce the importance of bacterial metabolic activities on the functioning of aquatic ecosystems (Pomeroy, 1974; Azam et al., 1983; Zehr, 2010) and call attention to the importance of biogeochemical processes developed by this community, such as respiration (Biddanda et al., 2001; Lennon and Pfaff, 2005) and the conversion of reduced forms of carbon into their biomass (Sherr and Sherr, 2002). Our findings highlighted that bacterial aerobic respiration was an important metabolic activity in the sediment of the reservoir and was fueled by the differential allochthonous OM delivery, as has been pointed out by the literature for lakes (Cole et al., 2000; Hanson et al., 2003; Kritzberg et al., 2004).

Evidence of allochthonous OC supporting metabolism in the water (Kritzberg et al., 2005; Cole et al., 2007; Karlsson et al., 2012) and in the sediment (Algesten et al., 2005; Kortelainen et al., 2006; Gudasz et al., 2010; Bai et al., 2012) of aquatic ecosystems has been reported in the literature, although the influence on OC mineralization in the sediments is still uncertain (Bai et al., 2012; Gudasz et al., 2012), especially for systems located in tropical areas and, in particular, man-made reservoirs (Battin et al., 2009; Tranvik et al., 2009). Furthermore, in carbon budget models the sediment is mainly seen as a site of carbon storage (Cole et al., 2007; Tranvik et al., 2009), and the amount of carbon that this compartment mineralizes back to the water and to the atmosphere is poorly considered. The carbon mineralized in the sediment, together with other sources of CO_2_ from the water column, is a significant carbon source to the atmosphere; especially if we take in account the area occupied by reservoirs in the landscape is increasing worldwide (World Commission on Dams [WCD], 2000). For instance, in the three different zones of the Manso reservoir, we found an OC mineralization average rate of roughly 100 mg C m^-^^2^ d^-^^1^. If the entire area of the reservoir (360 km^2^) is considered along 1 year, the carbon release from the sediment to the water is about 13 Gg C y^-^^1^.

In this study we found a positive relationship between the OC mineralization in the incubation cores and the *p*CO_2_ measurements made in the surface water of the Manso Reservoir (*r*^2^ = 0.73), pointing toward sediment OC mineralization being an important source of the CO_2_ emitted from the reservoir (**Figures 3D** and **4**), and toward a close link between sediment OC mineralization and the spatial variability in surface water *p*CO_2_. It is important to point out that the *p*CO_2_ measurements were made when the reservoir was not stratified, such that CO_2_ production in the sediments can leave an imprint in the *p*CO_2_ of the surface water; during water stratification periods, the carbon coming from the mineralization in the sediment may be trapped in the hypolimnion and may not contribute to the CO_2_ flux from the water to the atmosphere. In addition, it is important to highlight that the relationship between the OC mineralization in the sediment and the *p*CO_2_ in the surface water of the reservoir can also be regulated by other factors such as the CO_2_ saturation in the water, the wind speed in the area and the depth of the reservoir (Guérin et al., 2006), which can affect the contribution of the OC mineralization to the amount of CO_2_ emitted by the reservoir as a whole. Moreover, a study of five hydroelectric reservoirs in Brazil, including the Manso Reservoir (Roland et al., 2010), reported that *p*CO_2_ and CO_2_ emissions are also linked to spatial variations of the systems. For instance, their study shows that in general, considering all the reservoirs, the sites closest to the dam of the reservoir tend to have the lowest *p*CO_2_ saturations compared to other zones such as transition and river. By considering the carbon flux reported by previous study (352 mg C m^-^^2^ d^-^^1^; in Roland et al., 2010) and the average OC mineralization rate found in the sediments of the different zones of the Manso Reservoir (~100 mg C m^-^^2^ d^-^^1^), we estimate that the OC mineralization in the sediment can contribute up to 28% of the CO_2_ emitted to the atmosphere. Those findings reinforce the importance of the relation between the OC mineralization in the sediment and the CO_2_ emissions, but also show that this relation is shaped by other factors that need to be better understood in order to arrive at more accurate estimate.

In summary, we found that the spatial variability in OC mineralization rates in the sediment may be linked to differences in allochthonous OM supply to the sediments, and mirrored in both the spatial variability of surface water *p*CO_2_ and bacterial abundances in the sediment. The OC mineralization rates in the sediment contribute significantly to the CO_2_ emission from the water to the atmosphere. These results highlight that sediments should be considered in the assessment and management of carbon emissions from reservoirs to the atmosphere.

## Conflict of Interest Statement

The authors declare that the research was conducted in the absence of any commercial or financial relationships that could be construed as a potential conflict of interest.
